# Stratified lymph node yield thresholds after neoadjuvant immunochemotherapy: a surgical benchmark for survival in oral squamous cell carcinoma

**DOI:** 10.3389/fimmu.2026.1782877

**Published:** 2026-06-03

**Authors:** Shuai Yuan, Yunshuang Hu, Yalin Hou, Yunzhe Wei, Qigen Fang, Fei Liu

**Affiliations:** 1 Department of Stomatology, The First Affiliated Hospital of Zhengzhou University, Zhengzhou, China; 2 Department of Head and Neck, The Affiliated Cancer Hospital of Zhengzhou University & Henan Cancer Hospital, Zhengzhou, China

**Keywords:** lymph node yield, neoadjuvant immunotherapy, oral squamous cell carcinoma, surgical adequacy, survival outcomes

## Abstract

**Background:**

The adequacy of neck dissection following neoadjuvant immunotherapy combined with chemotherapy (NICT) for locally advanced oral squamous cell carcinoma (OSCC) lacks evidence-based benchmarks. This study aimed to define and validate extent-specific lymph node dissection (LND) adequacy thresholds associated with survival outcomes.

**Methods:**

In a development and validation study, the training cohort comprised 256 consecutive patients with locally advanced OSCC treated with NICT and surgery at Henan Cancer Hospital (2019-2022). An independent cohort of 199 patients from the First Affiliated Hospital of Zhengzhou University (2020-2022) served for validation. Patients were stratified by surgical extent: unilateral (Group Un) or bilateral (Group Bi) neck dissection. Optimal LND thresholds predictive of 3-year overall survival (OS) were determined using restricted cubic splines and maximally selected rank statistics. For Group Un, the threshold (LND-Un) was based on total lymph node yield; for Group Bi, the threshold (LND-Bi) was based on the average yield per side. Survival and safety outcomes were compared between patients meeting (“Adequate”) or not meeting (“Inadequate”) these thresholds.

**Results:**

The optimal thresholds were 20 total lymph nodes for unilateral dissections (LND-Un) and 18 nodes per side on average for bilateral dissections (LND-Bi). In the training cohort, inadequate LND was independently associated with worse OS (Group Un: adjusted hazard ratio [aHR] 2.42, 95% CI 1.47-3.99, p<0.001; Group Bi: aHR 2.29, 95% CI 1.20-4.37, p=0.012) and disease-free survival (DFS). Inadequate LND was also linked to a higher risk of major complications in Group Un (adjusted odds ratio 2.15, 95% CI 1.06-4.38, p=0.034). These findings were robustly validated in the external cohort, where the LND-based model demonstrated good discrimination (C-index for OS: 0.71), excellent calibration, and positive net benefit on decision curve analysis.

**Conclusion:**

This study establishes and validates stratified, surgical extent-specific thresholds for lymph node dissection adequacy after NICT in OSCC. Achieving a yield of ≥20 nodes in unilateral dissection or an average of ≥18 nodes per side in bilateral dissection is independently associated with significantly improved survival and an acceptable safety profile. These benchmarks provide a tangible, evidence-based guide for surgical quality assessment and decision-making in the post-NICT setting.

## Introduction

Oral squamous cell carcinoma (OSCC) represents a significant global health burden, accounting for the majority of head and neck malignancies. Despite advances in multidisciplinary management, the prognosis for patients with locally advanced disease remains suboptimal, with high rates of locoregional recurrence and distant metastasis (1). The integration of neoadjuvant immunotherapy combined with chemotherapy (NICT) has emerged as a promising paradigm shift, aiming to downstage tumors, eradicate micrometastatic disease, and improve long-term survival outcomes (2). Following NICT, curative-intent surgical resection, including comprehensive neck dissection, remains the cornerstone of definitive local-regional therapy (3). However, the extent and adequacy of this surgical intervention in the post-NICT setting are subjects of ongoing debate and lack standardized, evidence-based benchmarks.

The primary goal of neck dissection in OSCC is oncologic control through the eradication of nodal disease. The concept of lymph node yield (LNY) has been extensively studied in the context of upfront surgery for various solid tumors. In colorectal (4), gastric (5), and esophageal cancers (6), ample evidence supports that a higher LNY is associated with more accurate pathological staging, reduced stage migration, and improved survival, leading to the establishment of minimum nodal harvest thresholds in clinical guidelines. In head and neck cancer, similar principles are believed to apply. A more comprehensive lymphadenectomy may enhance staging accuracy by reducing the risk of missing occult metastases and potentially confer a survival benefit through the removal of micrometastatic deposits (7–9). Nevertheless, the optimal extent of nodal dissection must be balanced against the risk of increased morbidity, including shoulder dysfunction, spinal accessory nerve injury, chyle leak, and increased operative time.

This balance becomes even more complex and critically important in the context of NICT. NICT can induce significant pathological responses, including pathological complete response (pCR) and major pathological response (mPR), which alter the tumor microenvironment and nodal burden (10). A neck that has been downstaged or sterilized by effective systemic therapy may present a different anatomical and oncological landscape. The traditional surgical principles derived from the upfront surgery cohort may not be directly translatable. Performing an excessively radical dissection in a patient who has achieved a profound response could lead to unnecessary morbidity without additional oncologic benefit. Conversely, an inadequate dissection in a patient with residual resistant disease could compromise cure. Current clinical practice lacks specific guidelines for the extent of neck dissection following NICT. Decisions are often based on the pre-treatment clinical nodal stage, the surgeon’s assessment of response, and institutional tradition, leading to significant heterogeneity. Therefore, defining what constitutes an “adequate” lymph node dissection after NICT is a pressing, yet unanswered, clinical question.

To address these critical gaps in knowledge, our primary objective was to determine and validate stratified, surgical extent-specific thresholds for lymph node dissection adequacy. We hypothesized that such thresholds exist, are different for unilateral and bilateral neck dissections, and that achieving these benchmarks would be independently associated with improved survival and an acceptable safety profile.

## Methods

### Study design and ethical approval

The study utilized a development and validation design involving two independent cohorts: a training cohort for model development and a single-center external validation cohort for subsequent testing. The training cohort retrospectively analyzed patient data from Henan Cancer Hospital, while the external validation cohort comprised consecutive patients from the First Affiliated Hospital of Zhengzhou University. The research was conducted in full accordance with the Declaration of Helsinki, and the study protocol received approval from the institutional review boards of the two participating hospitals. Informed written consent was obtained from all patients.

### Study populations

The training cohort comprised consecutive patients with locally advanced OSCC (cT2N1-3 or T3/4N0 according to AJCC 8th edition) who underwent ≥ 2 cycles of NICT followed by curative-intent surgery (R0 resection) between January 2019 and December 2022. The validation cohort was assembled from consecutive patients treated at the First Affiliated Hospital of Zhengzhou University between January 2020 and December 2022 applying the same eligibility criteria. The inclusion criteria applied to both groups were: histologically confirmed, previously untreated, locally advanced disease; completion of the specified NICT regimen; radical R0 resection with neck dissection; age between 18 and 75 years; and the availability of complete clinical, pathological, and follow-up data. Patients were excluded if they presented with distant metastasis at diagnosis, had a history of other malignancies within five years, underwent non-curative resection (R1 or R2), or had incomplete neoadjuvant treatment or a major protocol violation.

### Treatment protocols

NICT combined a PD-1 inhibitor (pembrolizumab, toripalimab, or camrelizumab, 200 mg every three weeks) with platinum-based chemotherapy (75 mg/m² every three weeks). Surgical intervention was performed 4 to 6 weeks following the last NICT dose based on institutional protocol (3). The procedure included resection of the primary tumor with adequate margins tailored to its anatomical site, accompanied by unilateral or bilateral neck dissection based on post-NICT tumor location and initial nodal status; these dissections adhered to levels (I-V), and all specimens were submitted en bloc for pathological examination. Lymph node sample was manipulated using fat clearance technique. Subsequently, the decision to administer adjuvant radiotherapy with or without concurrent systemic therapy was determined by final pathological findings including extranodal extension, or residual nodal disease in conjunction with an assessment of the patient’s overall fitness.

### Data collection

Data were collected using standardized case report forms from electronic medical records. Key variables recorded encompassed patient demographics (age, sex), clinical history (smoking/alcohol status, BMI, tumor subsite, clinical TNM stage), treatment details (NICT regimen, number of cycles, type of neck dissection, operative time [incision-to-closure time]), and pathological data. The latter included pathological T and N staging (ypT, ypN) according to the AJCC 8th edition, total lymph nodes retrieved and number of positive nodes per anatomical level, and the presence of lymphovascular invasion (LVI) and perineural invasion (PNI). Treatment response was assessed histologically in the primary tumor using the Tumor Regression Grade (TRG) system, with major Pathological Response (mPR) defined as ≤10% residual viable tumor cells and Pathological Complete Response (pCR) defined as the absence of viable tumor cells in both the primary site and lymph nodes (11).

Outcome measures included overall survival (OS), defined as the time from surgery to death from any cause, and disease-free survival (DFS), defined as the time from surgery to recurrence or death. Follow-up was conducted every 3 to 6 months for the first 2 years and annually thereafter.

### Determination of lymph node dissection threshold

To address the heterogeneity in surgical extent between unilateral and bilateral neck dissections, the analysis of lymph node dissection (LND) was stratified accordingly. The training cohort was divided into two mutually exclusive groups: Group Un (patients undergoing Unilateral Neck Dissection) and Group Bi (those undergoing Bilateral Neck Dissection). To derive clinically relevant thresholds, the total number of retrieved lymph nodes was analyzed for Group Un, while for Group Bi the average nodal yield per side (calculated as total LND divided by 2) was used to allow for a standardized comparison of intensity. For each group, an optimal cut-off value predictive of 3-year OS was determined using restricted cubic splines and maximally selected rank statistics, yielding two distinct thresholds designated LND-Un and LND-Bi. Finally, for survival analysis, patients in the training cohort were categorized based on these thresholds: those in Group Un were classified as having “Adequate-Un LND” if their total LND was ≥ LND-Un, or otherwise as “Inadequate-Un LND”; similarly, patients in Group Bi were classified as having “Adequate-Bi LND” if their average LND per side was ≥ LND-Bi, or otherwise as “Inadequate-Bi LND”.

### Statistical analysis

Descriptive statistics were used to summarize patient characteristics. Categorical variables are presented as frequencies (percentages) and compared using the Chi-square or Fisher’s exact test. Continuous variables are reported as medians with interquartile ranges (IQR) and compared using the Mann–Whitney U test.

Survival outcomes were analyzed separately for patients undergoing unilateral (Group Un) and bilateral (Group Bi) neck dissection. Kaplan–Meier curves for OS and DFS were plotted, and differences between “Adequate” and “Inadequate” LND groups were compared using the log-rank test. Univariable and multivariable Cox proportional hazards models were fitted for each surgical stratum to identify independent prognostic factors. Covariates included age, ypT, ypN, mPR, LVI, PNI, adjuvant therapy, and major postoperative complications (Clavien–Dindo grade ≥ III). The LND adequacy variable was retained in all final models.

Postoperative complications were compared between Adequate and Inadequate LND groups within each surgical stratum. The primary safety endpoint was the incidence of major complications (Clavien–Dindo grade ≥ III). Rates were compared using Chi-square/Fisher’s exact tests, with adjusted analysis via multivariable logistic regression for age, BMI, operative time, and other relevant factors.

For external validation, the surgical stratification and LND thresholds (LND-Un, LND-Bi) derived from the training cohort were directly applied to the validation cohort. Patients were categorized accordingly, and Kaplan–Meier analyses for OS/DFS and complication comparisons were performed. In addition, the predictive performance of the LND-based risk classification was formally evaluated in the validation cohort using established metrics: discrimination was assessed using Harrell’s concordance index (C-index) for time-to-event outcomes; calibration was evaluated by plotting predicted versus observed 1-year and 3-year survival probabilities, with smoothed calibration curves generated using loess regression; clinical utility was explored through decision curve analysis (DCA), quantifying net benefit across a range of risk thresholds for clinical decision-making.

Sensitivity analyses included: pooled multivariable Cox regression of the entire training cohort with “surgical extent” as a covariate; re-analysis using total LND (instead of average) for Group Bi. Missing data >5% were handled via multiple imputation, with complete-case analysis as a sensitivity check. A two-sided p-value < 0.05 was considered statistically significant. Analyses were performed using R (v4.3.0) and SPSS (v26.0).

## Results

### Baseline data

In total, there were 256 patients in training group and 199 patients in validation cohort. Both cohorts comprised predominantly older males (median age 62 and 60 years; 72.3% and 71.4% male) with a low median BMI (approximately 20.1–20.5 kg/m²). A majority of patients had a history of smoking (62.9% and 59.8%) and over half reported current or former alcohol use. The most common tumor subsite was the tongue (43.0% and 42.7%), followed by the floor of mouth and buccal mucosa. Clinically, the cohorts presented with locally advanced disease, with cT3/T4 stages in 60.2% and 60.8% of patients, and node-positive disease (cN1-3) in 65.6% and 66.3%, respectively. Most received NICT (median 3 cycles) followed by surgery and adjuvant radiotherapy, with approximately half receiving concurrent chemoradiotherapy. Critically, no statistically significant differences were observed between the two cohorts across any demographic, clinical, pathological, or treatment-related variable (all p > 0.05). (**Supplementary Table 1**).

### Determination of the lymph node dissection thresholds

**Figure 1:**
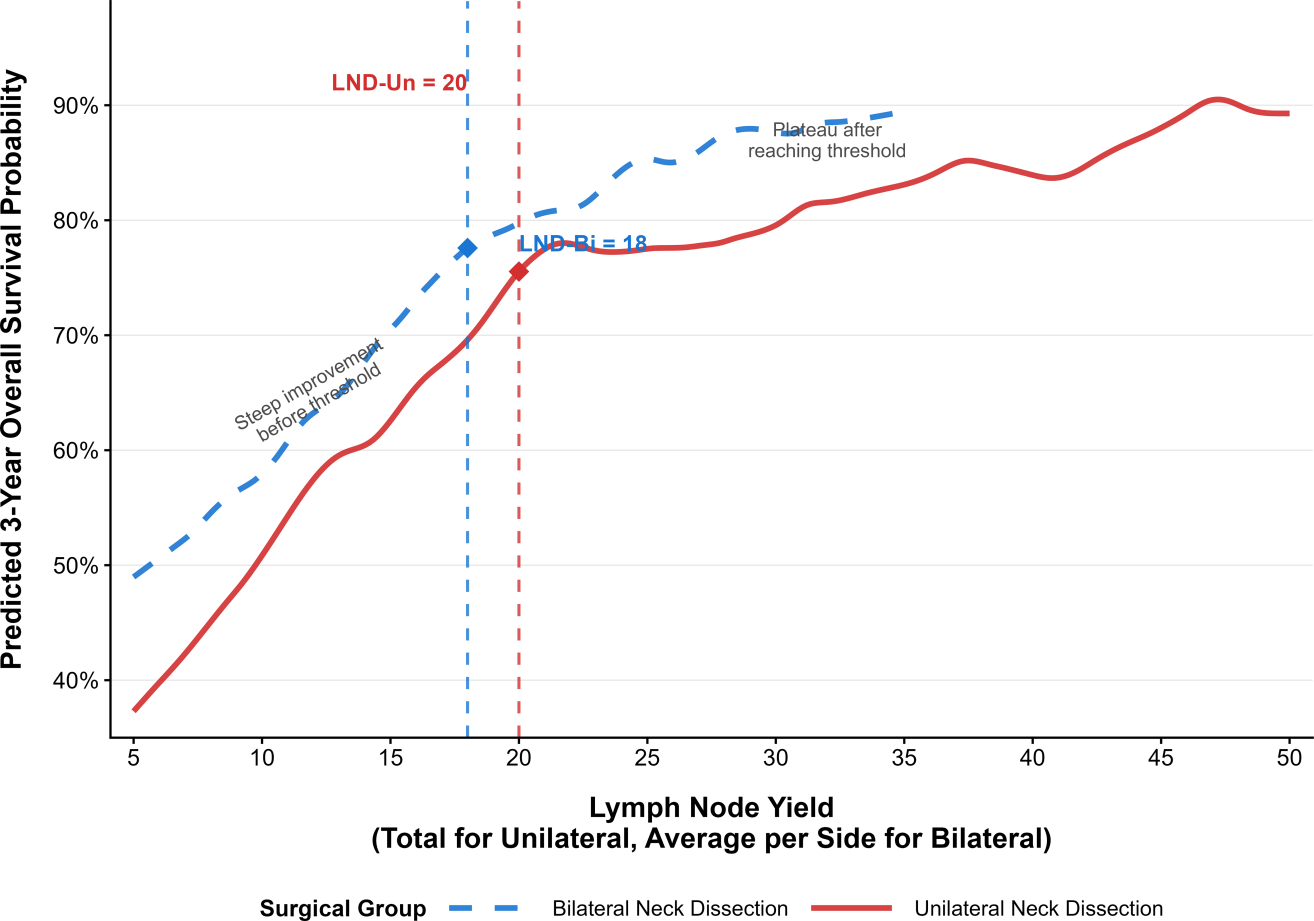
Association between lymph node yield and predicted 3-year OS probability according to surgical extent.

**Figure 2:**
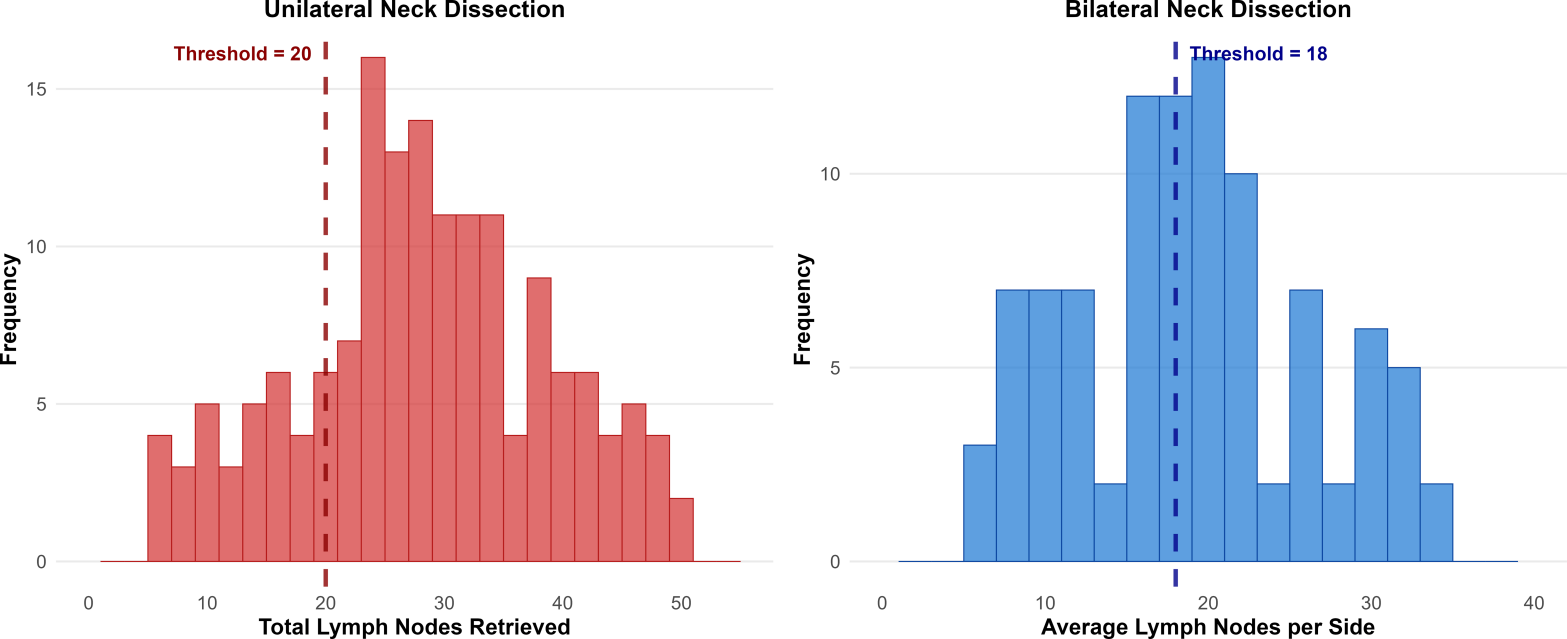
Lymph node yield distribution and threshold achievement rates.

For the unilateral dissection cohort (Group Un, n=158), the analysis revealed an optimal cut-off of 20 total LNs (LND-Un; 95%CI: 18-23), with patients below this threshold demonstrating significantly inferior survival outcomes. For the bilateral dissection cohort (Group Bi, n=98), the optimal threshold was 18LNs per side on average (LND-Bi; 95% CI: 16-20), derived by dividing the total nodal yield by two to standardize comparison across sides. The relationship between LNY and predicted 3-year OS probability exhibited a characteristic nonlinear pattern: a steep, approximately linear increase in survival probability up to each threshold, followed by a plateau phase where additional nodal retrieval conferred diminishing marginal benefit (**Figure 1**). The restricted cubic spline curves demonstrated good fit (adjusted R²=0.76 for unilateral, 0.72 for bilateral), with the identified thresholds corresponding to inflection points where the second derivative of the survival function approached zero. Validation of these thresholds against the actual distribution of lymph node yield (**Figure 2**) confirmed their clinical relevance, as both cut-offs resided within the interquartile ranges of their respective distributions (LND-Un within 22-35; LND-Bi within 14-23). Consequently, 68.4% of unilateral patients (108/158) and 63.3% of bilateral patients (62/98) met or exceeded these benchmarks.

### Prognosis

**Figure 3:**
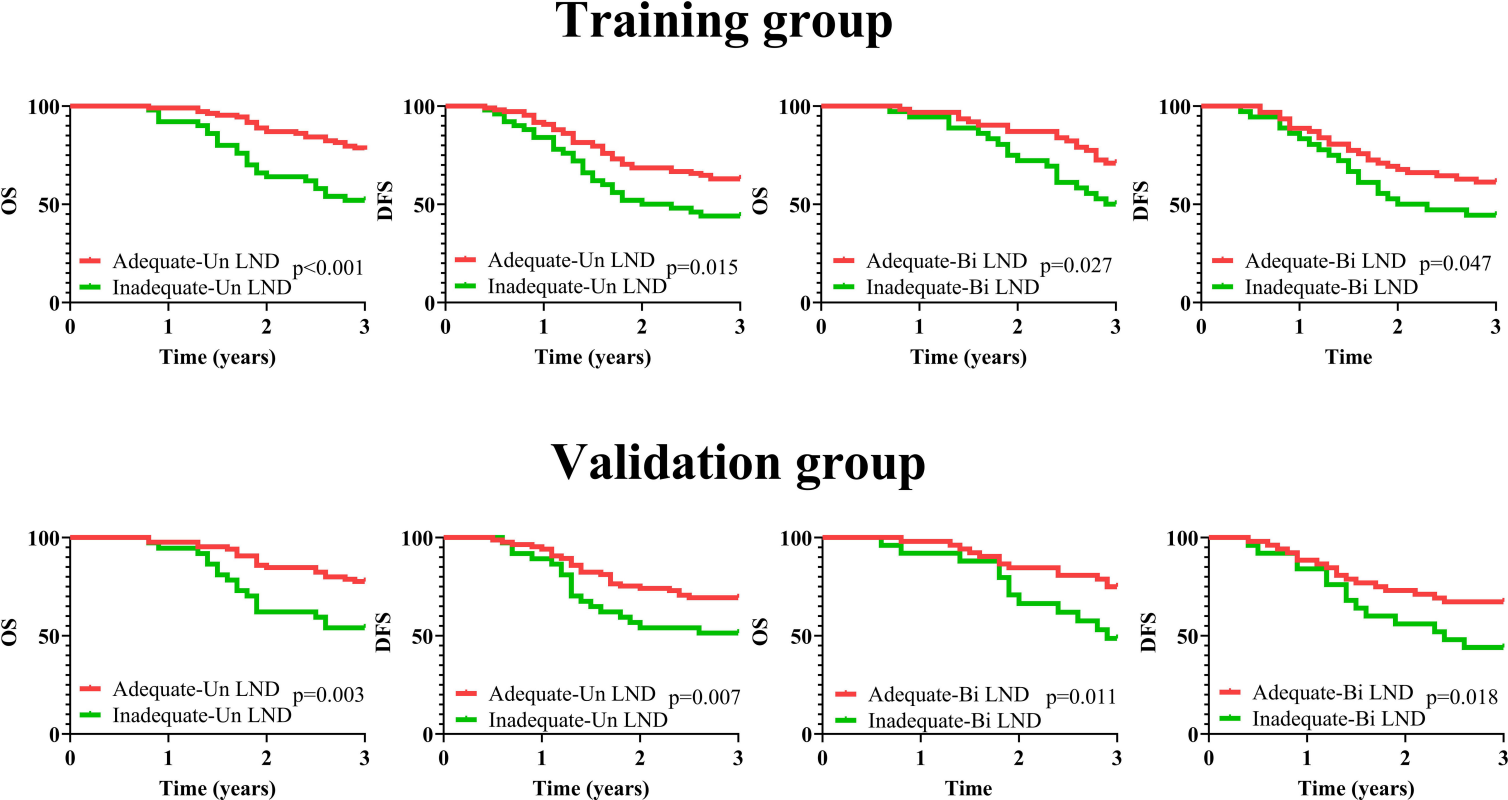
Survival analysis of the training and validation cohorts stratified by LND adequacy.

The median follow-up time for the entire training cohort was 36.2 months (IQR: 24.5–48.1 months). During this period, 84 of 256 patients (32.8%) experienced OS event, and 112 patients (43.8%) experienced DFS event. When stratified by surgical extent, patients in Group Un had a median follow-up of 35.8 months (IQR: 24.1–47.5 months), during which 48 OS events (30.4%) and 68 DFS events (43.0%) were observed. Within this group, the Adequate-Un LND subgroup experienced 24 OS events (22.2%) and 40 DFS events (37.0%), corresponding to 3-year OS and DFS rates of 78.2% and 70.1%, respectively. In contrast, the Inadequate-Un LND subgroup experienced 24 OS events (48.0%) and 28 DFS events (56.0%), with significantly lower 3-year OS and DFS rates of 52.4% and 48.9% (**Figure 3**). For patients in Group Bi, the median follow-up was 36.7 months (IQR: 25.2–49.0 months), with 36 OS events (36.7%) and 44 DFS events (44.9%). Within this group, the Adequate-Bi LND subgroup experienced 18 OS events (29.0%) and 24 DFS events (38.7%), achieving 3-year OS and DFS rates of 75.8% and 67.7%. The Inadequate-Bi LND subgroup experienced 18 OS events (50.0%) and 20 DFS events (55.6%), with substantially lower 3-year OS and DFS rates of 52.1% and 45.6% (**Figure 3**).

For patients in Group Un, inadequate LND was significantly associated with worse OS (aHR 2.42, 95%CI 1.47-3.99, p<0.001). Other independent prognostic factors for OS in this group included pathological T stage (ypT3/4, aHR 1.65, p=0.034), pathological N stage (ypN+, aHR 1.88, p=0.006), LVI (aHR 2.10, p=0.044), and pathological response (non-mPR, aHR 1.78, p=0.012). (**Table 1**).

**Table 1:** Cox proportional hazards analysis for OS in group Un (Unilateral).

Variable	Category	Univariable		Multivariable	
		HR (95% CI)	p	aHR (95% CI)	p
LND Adequacy	Inadequate-Un LND (<20)	2.85 (1.75–4.65)	<0.001	2.42 (1.47–3.99)	<0.001
Age	≥62 vs. <62 years	1.32 (0.85–2.06)	0.216	1.28 (0.81–2.01)	0.288
Sex	Male vs. Female	1.18 (0.72–1.93)	0.513	1.12 (0.67–1.86)	0.667
BMI	≥20.1 vs. <20.1 kg/m²	0.89 (0.58–1.37)	0.601	0.92 (0.59–1.43)	0.702
Smoking Status	Current/Former vs. Never	1.25 (0.79–1.98)	0.347	1.21 (0.76–1.93)	0.427
Alcohol Status	Current/Former vs. Never	1.17 (0.75–1.83)	0.487	1.14 (0.73–1.79)	0.568
Tumor Subsite	Non-tongue vs. Tongue	1.29 (0.83–2.01)	0.257	1.25 (0.80–1.96)	0.328
Clinical T Stage	cT3/4 vs. cT2	1.45 (0.93–2.26)	0.104	1.38 (0.88–2.16)	0.162
Clinical N Stage	cN+ vs. cN0	1.52 (0.94–2.46)	0.089	1.46 (0.89–2.38)	0.132
Pathological T Stage	ypT3/4 vs. ypT0-2	1.89 (1.21–2.95)	0.005	1.65 (1.04–2.62)	0.034
Pathological N Stage	ypN+ vs. ypN0	2.14 (1.38–3.31)	0.001	1.88 (1.20–2.95)	0.006
LVI	Present vs. Absent	2.45 (1.21–4.96)	0.013	2.10 (1.02–4.32)	0.044
PNI	Present vs. Absent	1.78 (0.82–3.87)	0.145	1.56 (0.71–3.43)	0.271
Pathological Response	Non-mPR vs. mPR/pCR	2.01 (1.30–3.11)	0.002	1.78 (1.14–2.78)	0.012
Level IV/V resect	No vs. Yes	1.78 (0.74-2.98)	0.187	1.88 (0.65-3.15)	0.219
NICT Cycles	≥3 vs. <3	0.94 (0.61–1.45)	0.779	0.97 (0.62–1.51)	0.887
Adjuvant Therapy	CRT vs. RT	0.76 (0.49–1.18)	0.220	0.80 (0.51–1.25)	0.327
Major Complications	Clavien-Dindo ≥III vs. <III	1.65 (0.91–3.00)	0.101	1.48 (0.81–2.73)	0.204

HR, Hazard Ratio; aHR, adjusted Hazard Ratio; Bold indicates statistical significance (p<0.05).

For patients in Group Bi, inadequate LND was independently associated with poorer OS (aHR 2.29, 95% CI 1.2-4.37, p=0.012). Additional independent predictors of OS in this cohort included pathological N stage (aHR 2.05, p=0.020), LVI (aHR 2.41, p=0.047), and pathological response (aHR 1.89, p=0.039) (**Table 2**).

**Table 2:** Cox proportional hazards analysis for OS in group Bi (Bilateral).

Variable	Category	Univariable		Multivariable	
		HR (95% CI)	p	aHR (95% CI)	p
LND Adequacy	Inadequate-Bi LND (<18/side)	2.61 (1.38–4.93)	0.003	2.29 (1.20–4.37)	0.012
Age	≥60 vs. <60 years	1.41 (0.78–2.55)	0.260	1.35 (0.73–2.48)	0.340
Sex	Male vs. Female	1.31 (0.66–2.61)	0.442	1.28 (0.64–2.57)	0.488
BMI	≥20.5 vs. <20.5 kg/m²	0.82 (0.45–1.48)	0.504	0.85 (0.47–1.55)	0.597
Smoking Status	Current/Former vs. Never	1.42 (0.77–2.61)	0.262	1.38 (0.75–2.55)	0.303
Alcohol Status	Current/Former vs. Never	1.23 (0.68–2.23)	0.491	1.20 (0.66–2.19)	0.551
Tumor Subsite	Non-tongue vs. Tongue	1.48 (0.81–2.69)	0.203	1.42 (0.78–2.60)	0.255
Clinical T Stage	cT3/4 vs. cT2	1.58 (0.87–2.87)	0.132	1.52 (0.83–2.78)	0.174
Clinical N Stage	cN+ vs. cN0	1.65 (0.86–3.16)	0.132	1.58 (0.82–3.05)	0.170
Pathological T Stage	ypT3/4 vs. ypT0-2	1.96 (1.08–3.55)	0.026	1.74 (0.95–3.20)	0.074
Pathological N Stage	ypN+ vs. ypN0	2.33 (1.29–4.21)	0.005	2.05 (1.12–3.76)	0.020
LVI	Present vs. Absent	2.88 (1.23–6.75)	0.015	2.41 (1.01–5.75)	0.047
PNI	Present vs. Absent	1.92 (0.73–5.05)	0.185	1.68 (0.63–4.49)	0.300
Pathological Response	Non-mPR vs. mPR/pCR	2.12 (1.17–3.83)	0.013	1.89 (1.03–3.45)	0.039
Level IV/V resect	No vs. Yes	1.64 (0.72-2.73)	0.214	1.91 (0.68-3.13)	0.188
NICT Cycles	≥3 vs. <3	0.88 (0.48–1.60)	0.669	0.91 (0.50–1.66)	0.758
Adjuvant Therapy	CRT vs. RT	0.69 (0.38–1.25)	0.220	0.72 (0.39–1.33)	0.291
Major Complications	Clavien-Dindo ≥III vs. <III	1.89 (0.91–3.93)	0.088	1.72 (0.82–3.62)	0.151

HR, Hazard Ratio; aHR, adjusted Hazard Ratio; Bold indicates statistical significance (p<0.05).

In Group Un, inadequate LND was significantly associated with worse DFS (aHR 2.18, 95% CI 1.35-3.52, p=0.001). Other independent prognostic factors for DFS included pathological T stage (ypT3/4, aHR 1.62, p=0.027), pathological N stage (ypN+, aHR 1.82, p=0.006), LVI (aHR 2.01, p=0.047), and pathological response (non-mPR, aHR 1.70, p=0.015) (**Supplementary Table 2**).

In Group Bi, inadequate LND was independently associated with poorer DFS (aHR 2.05, 95% CI 1.10-3.82, p=0.024). Pathological N stage (ypN+, aHR 1.95, p=0.026) remained the only additional independent predictor of DFS in this cohort (**Supplementary Table 3**).

### Safety

In Group Un, patients with Inadequate-Un LND experienced a significantly higher rate of major complications compared to those with Adequate-Un LND (20.0% [10/50] vs. 11.1% [12/108]; p=0.041). In contrast, within Group Bi, the complication rates did not differ significantly between Inadequate-Bi LND and Adequate-Bi LND (19.4% [7/36] vs. 16.1% [10/62]; p=0.620). Subsequently, after adjusting for potential confounders including age, sex, BMI, smoking and alcohol status, operative time, pathological T and N stage, LVI, PNI, and extent of neck dissection. In Group Un, Inadequate LND remained independently associated with a significantly increased risk of major complications (aOR 2.15, 95% CI 1.06-4.38, p=0.034). Additional independent predictors included older age (aOR 1.04 per 3-year, p=0.049) and longer operative time (aOR 1.13 per 30-minute increase, p=0.014). Conversely, in Group Bi, LND adequacy was not independently associated with complication risk (aOR 1.22, 95% CI 0.56-2.66, p=0.618), with operative time being the sole significant predictor (aOR 1.19 per 30-minute increase, p=0.008) (**Table 3**).

**Table 3:** Multivariable logistic regression for major complications (Clavien–Dindo ≥ III) by Surgical Extent.

Variable	Category	Group Un		Group Bi	
		aOR (95% CI)	p	aOR (95% CI)	p
LND Adequacy	Inadequate LND	**2.15 (1.06–4.38)**	**0.034**	1.22 (0.56–2.66)	0.618
	Adequate LND (Ref.)	1.00	–	1.00	–
Age	Per 3-year increase	**1.04 (1.00–1.08)**	**0.049**	1.02 (0.97–1.08)	0.398
Sex	Male	1.18 (0.60–2.32)	0.632	1.31 (0.61–2.79)	0.487
	Female (Ref.)	1.00	–	1.00	–
BMI	Per 1 kg/m² increase	0.96 (0.87–1.06)	0.432	0.98 (0.88–1.10)	0.744
Smoking Status	Current/Former	1.25 (0.70–2.23)	0.451	1.40 (0.71–2.77)	0.335
	Never (Ref.)	1.00	–	1.00	–
Alcohol Status	Current/Former	1.12 (0.63–1.99)	0.699	1.18 (0.60–2.31)	0.637
	Never (Ref.)	1.00	–	1.00	–
Operative Time	Per 30-minute increase	**1.13 (1.03–1.24)**	**0.014**	**1.19 (1.05–1.36)**	**0.008**
pT Stage	ypT3/4	1.48 (0.80–2.74)	0.210	1.28 (0.64–2.57)	0.485
	ypT0-2 (Ref.)	1.00	–	1.00	–
pN Stage	ypN+	1.18 (0.66–2.10)	0.581	1.39 (0.72–2.68)	0.329
	ypN0 (Ref.)	1.00	–	1.00	–
LVI	Present	1.45 (0.68–3.10)	0.340	1.52 (0.70–3.31)	0.296
	Absent (Ref.)	1.00	–	1.00	–
PNI	Present	1.33 (0.59–3.00)	0.495	1.41 (0.64–3.10)	0.396
	Absent (Ref.)	1.00	–	1.00	–
Level IV/V resect	No	0.86 (0.48–1.54)	0.611	0.92 (0.47–1.82)	0.815
	Yes (Ref.)	1.00	–	1.00	–

aOR, adjusted odds ratio; CI, confidence interval; Ref., reference category; LVI, lymphovascular invasion; PNI, perineural invasion.

Bold indicates statistical significance (p<0.05).

### Validation

**Figure 4:**
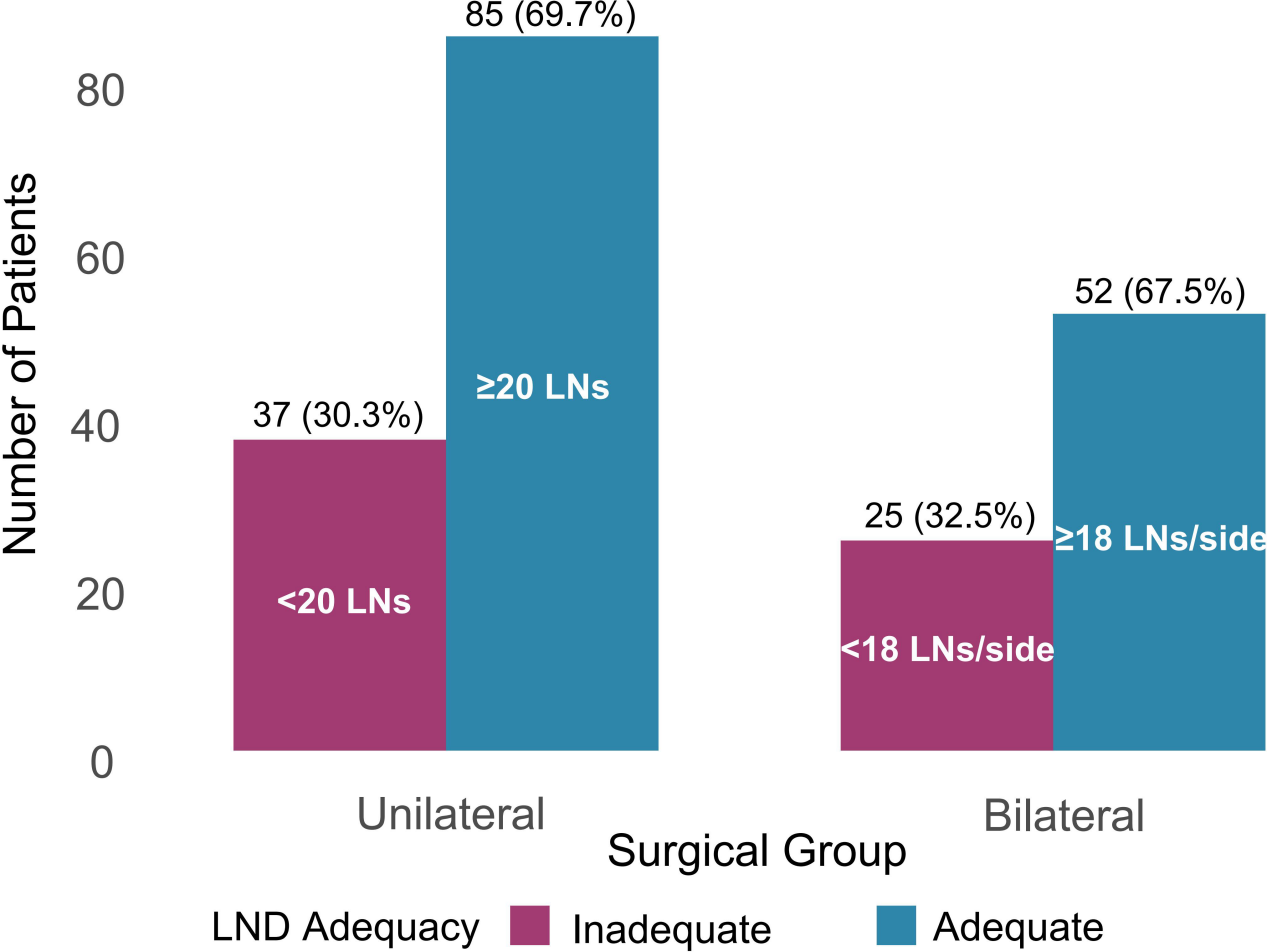
LND adequacy distribution in the validation cohort.

In the independent validation cohort of 199 patients, among the 122 patients in Group Un, 85 patients (69.7%) were classified as having Adequate-Un LND, while 37 patients (30.3%) were classified as Inadequate-Un LND. Among the 77 patients in Group Bi, 52 patients (67.5%) were classified as having Adequate-Bi LND, and 25 patients (32.5%) as Inadequate-Bi LND (**Figure 4**).

Kaplan-Meier survival analysis of the validation cohort confirmed the prognostic significance of LND adequacy thresholds (**Figure 4**). For Group Un, patients with Adequate-Un LND demonstrated significantly superior 3-year OS (77.1% vs. 53.2%, log-rank p=0.003) and DFS (68.9% vs. 46.5%, log-rank p=0.007) compared to those with Inadequate-Un LND. Similarly, for Group Bi, patients with Adequate-Bi LND showed significantly better 3-year OS (74.6% vs. 51.8%, log-rank p=0.011) and DFS (66.3% vs. 43.1%, log-rank p=0.018) than those with Inadequate-Bi LND (**Figure 3**).

**Figure 5:**
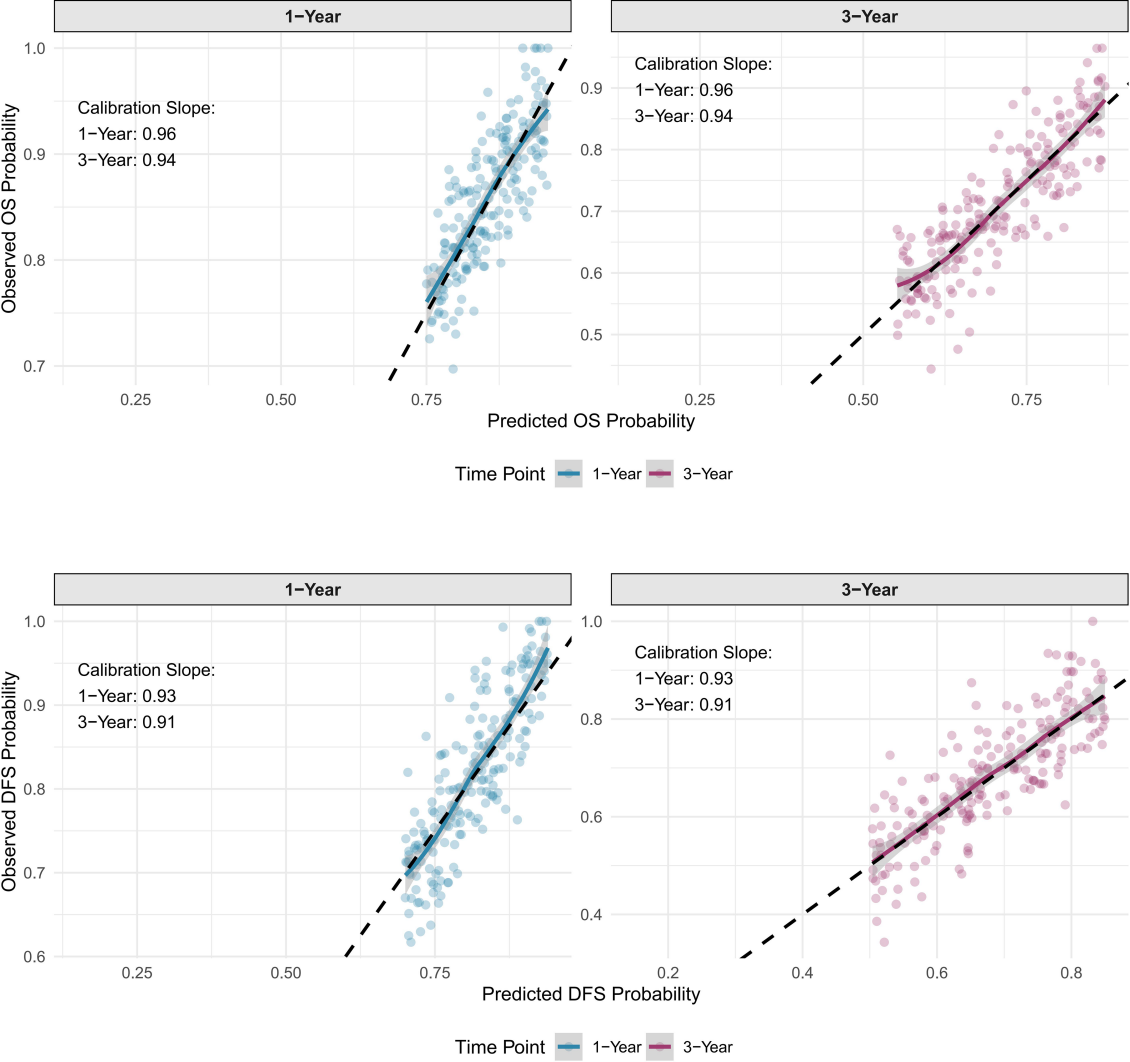
Calibration plots for the LND-based prediction model.

Predictive performance analysis demonstrated that the LND-based model maintained good discrimination in the validation cohort. The C-index was 0.71 (95% CI: 0.65-0.77) for OS and 0.68 (95% CI: 0.62-0.74) for DFS, indicating moderate to good discriminatory ability. Calibration analysis revealed excellent agreement between predicted and observed survival probabilities (**Figure 5**). The calibration curves for both 1-year and 3-year OS and DFS closely aligned with the 45-degree line of perfect agreement, with calibration slopes ranging from 0.91 to 0.96 and intercepts near zero (0.02-0.04), confirming neither systematic overestimation nor underestimation of survival probabilities. Brier scores ranged from 0.08 to 0.14 across different time points, further validating the model’s predictive accuracy.

**Figure 6:**
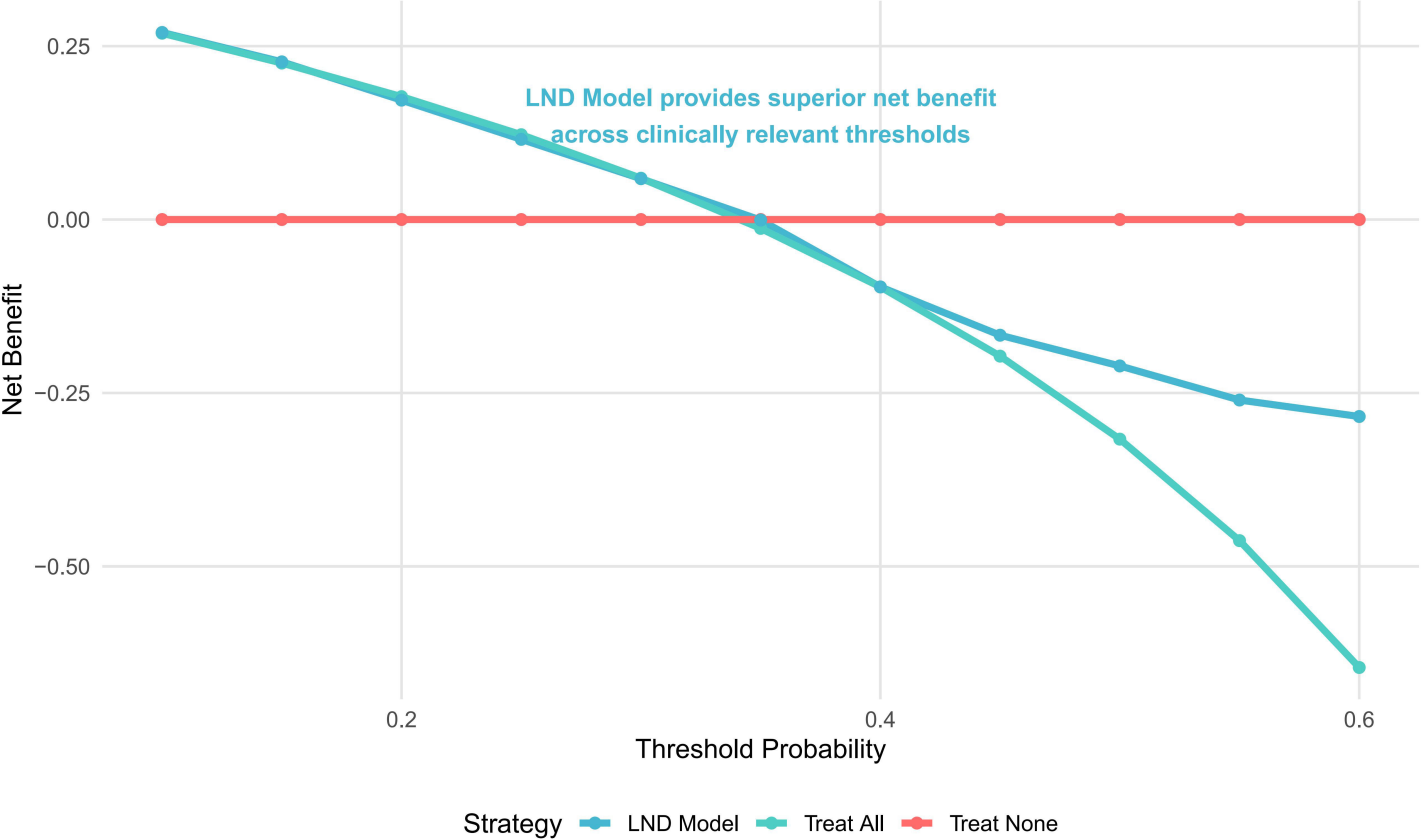
Decision curve analysis of the LND-based risk classification for overall survival.

Decision curve analysis demonstrated the clinical utility of the LND-based risk classification for OS. Across a clinically relevant range of threshold probabilities (10-60%), the LND model provided superior net benefit compared to both “treat-all” and “treat-none” strategies. The maximal net benefit advantage was observed at threshold probabilities of approximately 40%, where the LND model outperformed the “treat-all” strategy by 15% in net benefit, indicating its potential value for guiding clinical decision-making regarding adjuvant therapy intensification (**Figure 6**).

### Sensitivity analysis

First, a pooled multivariable Cox regression of the entire training cohort (n=256) incorporating surgical extent (unilateral vs. bilateral) as a covariate confirmed that LND adequacy remained a strong, independent predictor of both OS (aHR 2.36, 95% CI 1.61-3.45, p<0.001) and DFS (aHR 2.12, 95% CI 1.47-3.06, p<0.001), while surgical extent itself was not significantly associated with survival outcomes (p>0.05). Second, re-analyzing the bilateral dissection cohort using total lymph node yield instead of the average per side yielded an optimal threshold of 36 total LNs, effectively double the per-side benchmark. Patients with total LND <36 demonstrated significantly inferior OS (aHR 2.18, 95% CI 1.14-4.16, p=0.018) and DFS (aHR 2.01, 95% CI 1.08-3.74, p=0.027), corroborating the clinical relevance of the original per-side threshold. Third, comparison of models derived from complete-case analysis and multiple imputation for handling missing data revealed nearly identical HRs and CIs for LND adequacy in both surgical groups, with all p-values remaining significant and calibration metrics virtually unchanged (**Supplementary Table 4**).

## Discussion

This development and validation study establishes and confirms stratified, surgical extent-specific LNY thresholds for neck dissection following NICT in locally advanced OSCC. We identified an optimal yield of ≥20 total LNs for unilateral dissections and an average of ≥18 nodes per side for bilateral dissections, benchmarks that are independently associated with significantly improved overall and disease-free survival. The robust validation across an external cohort, supported by strong model discrimination, excellent calibration, and positive net benefit on decision curve analysis, underscores the clinical relevance and potential utility of these thresholds as evidence-based surgical quality metrics in the post-NICT setting.

The prognostic significance of LNY in OSCC remains inconsistent across contemporary studies. Several investigations have proposed specific LNY thresholds associated with survival outcomes, yet findings vary widely. A Canadian single-center study (12) identified an LNY >15 as significantly correlated with improved DFS, reduced locoregional recurrence, and lower distant metastasis rates. Similarly, a Danish nationwide cohort (13) reported that an LNY of 21 was linked to better OS but only among node-positive patients, with no benefit observed in node-negative cases. In contrast, a multicenter analysis using advanced causal inference methods (14) found no survival advantage associated with higher LNY in either pN0 or pN+ OSCC patients, challenging the assumption that greater nodal harvest improves outcomes. Further complicating the picture, one additional study failed to validate LNY as an independent prognostic factor (15) and reported non-significant associations in multivariable models after adjusting for key confounders such as extranodal extension, tumor depth, and adjuvant therapy. Notably, some of these studies selectively excluded LNY from final regression models due to lack of statistical significance, raising concerns about reporting bias. Moreover, methodological differences including variations in neck dissection extent (selective vs. comprehensive), pathological processing protocols, and patient selection likely contribute to the divergent conclusions. Collectively, these six studies highlight a lack of consensus on a clinically meaningful LNY benchmark in OSCC. Given the increasing use of LNY as a surgical quality indicator, this uncertainty underscores the necessity of context-specific research. The introduction of NICT for OSCC fundamentally reshapes the tumor microenvironment, induces nodal regression, and alters lymphatic architecture, potentially reducing the number of retrievable nodes independent of surgical quality. Consequently, conventional LNY thresholds derived largely from treatment-naïve or adjuvant-only cohorts may no longer reflect oncologic adequacy or predict outcomes in the NICT setting, necessitating new, context-specific benchmarks.

LNY has been increasingly explored across solid tumors in the context of NICT. In esophageal squamous cell carcinoma, studies have identified optimal LND counts, such as ≥19 or 48 nodes, as associated with improved survival and accurate nodal staging following NICT (16, 17). Similarly, in muscle-invasive bladder cancer, adequate LND after neoadjuvant chemotherapy has been linked to better oncologic outcomes, though its interaction with modern systemic therapies remains incompletely defined (18). In rectal cancer, a LN count >11 was found critical for DFS in stage III patients post-chemoradiation, yet the effect varied by nodal status (19). Even in breast cancer, neoadjuvant regimens have been shown to reduce axillary lymph node retrieval, challenging traditional benchmarks for adequacy (20). Despite these advances, consensus on clinically meaningful LNY thresholds remains elusive, compounded by differences in surgical extent, pathological protocols, and tumor biology. Notably, none studies address OSCC, where the anatomical constraints and lymphatic drainage patterns differ substantially. To our knowledge, this is the first study to define and validate surgical extent, specific LNY thresholds ≥20 nodes for unilateral and ≥18 per side for bilateral neck dissections, in locally advanced OSCC following NICT.

The identified thresholds carry substantial biological and clinical relevance. Achieving these benchmarks ensures adequate staging accuracy by reducing the risk of missing occult nodal disease, which is critical in the post-NICT setting where pathological downstaging is common. A comprehensive nodal harvest facilitates the detection of residual micrometastatic deposits that might otherwise remain undetected, thereby enabling more accurate risk stratification and informing decisions regarding adjuvant therapy. Moreover, meeting these thresholds likely reflects the surgical removal of immunologically active nodal tissue that may harbor residual resistant clones, thereby reducing the risk of locoregional recurrence. Moreover, the observed plateau effect in the spline curves suggests that once an adequate oncologic dissection is achieved, additional nodal retrieval confers diminishing marginal returns. This phenomenon may be attributed to the fact that, after a certain point, further lymph node harvest primarily represents non-disease-bearing tissue rather than additional metastatic foci. In the context of NICT, where significant nodal regression occurs, the residual nodal burden is often limited; thus, retrieving nodes beyond the threshold may not translate into additional survival benefit while potentially increasing operative morbidity. Interesting, the bilateral dissection threshold is numerically lower than the unilateral threshold, yet both represent a comparable intensity of dissection per operated side. This difference likely reflects anatomical and procedural realities: bilateral dissections often involve more comprehensive clearance of central and contralateral levels, which may yield relatively fewer nodes per side due to anatomical constraints and the distribution of lymphatic tissue. Conversely, unilateral dissections focused on the ipsilateral neck can retrieve a higher absolute number from levels I-V, justifying a slightly higher total threshold. Both thresholds, therefore, represent surgically meaningful targets that balance oncologic adequacy with procedural feasibility and safety.

LNY in OSCC is shaped by a confluence of surgeon-, pathologist-, tumor-, and patient-related factors. Crucially, variability in pathological processing including the specific methods used for lymph node retrieval (e.g., visual inspection and palpation versus the use of chemical fat clearance techniques) can significantly influence the final nodal count, completely independent of the actual surgical yield. Our results underscore the oncologic relevance of adequate nodal harvest in this novel therapeutic context and align with prior literature indicating that higher LNY often diverges in its specificity to the post-NICT setting, where conventional thresholds may no longer apply due to therapy-induced nodal regression and altered lymphatic architecture. Aweeda et al. (9) affirmed that surgical extent, surgeon experience, and academic treatment setting positively influence LNY. However, unlike variation in LNY primarily to technical or pathological factors, prior literature highlighted the critical role of intrinsic patient characteristics: advanced age, low BMI, and extranodal extension were independently associated with lower normalized lymph node counts, suggesting that host immune status and tumor aggressiveness significantly modulate nodal retrieval (21). These insights collectively indicate that LNY is not merely a surrogate of surgical quality but a complex biomarker reflecting the interplay between tumor biology, host immunity, and therapeutic history, particularly in the emerging era of NICT, where redefining adequacy thresholds is essential for accurate staging and optimal adjuvant decision-making.

An important issue is whether a higher LNY is associated with an increased risk of major complications. Previous research has highlighted various factors influencing complication rates: Mak et al. (22) reported pooled complication rates including 7.1% for surgical site infection and 5.2% for hemorrhage, but emphasized high heterogeneity and inconsistent methodological quality across studies; Mrosk et al. (23) found an overall 12.6% complication rate in OSCC patients, noting increased morbidity with more extensive (e.g., bilateral or modified radical) dissections; and Campbell et al. (24), using a large national database, identified a U-shaped relationship between surgeon volume and complications, with mid-volume surgeons having the highest rates (15.8%), underscoring the role of surgical experience and case complexity. In contrast, our study uniquely links complication risk directly to the adequacy of LND, showing that in patients undergoing unilateral neck dissection, those with inadequate LND experienced significantly higher major complication rates, but the difference was not observed in the bilateral group. Rather, an inadequate LNY likely serves as a surrogate marker for a technically hostile surgical field. NICT can induce profound local tissue reactions, including severe desmoplasia, dense fibrosis, and the obliteration of normal anatomical planes. In such scenarios, surgeons may intentionally limit the radicality of the dissection, resulting in a lower nodal yield, to mitigate the risk of catastrophic vascular or cranial nerve injury. Simultaneously, operating within this fibrotic and distorted anatomy inherently increases the likelihood of intraoperative bleeding, prolonged operative times, and subsequent wound complications. Furthermore, a low yield may reflect cases with bulky, treatment-resistant residual disease that infiltrates surrounding structures, making the procedure inherently more morbid. The absence of this association in the bilateral group may be due to the generally higher baseline morbidity and complexity of bilateral procedures, which could mask the specific impact of these localized technical challenges.

This study has several limitations that warrant consideration. First, its retrospective, non-randomized design inherently carries risks of unmeasured confounding and selection bias. Second, although the use of two independent cohorts from high-volume centers enhances internal validity, the patient population was exclusively Chinese. This limits the direct extrapolation of the proposed thresholds to other ethnic or geographic groups, particularly concerning Western surgical and pathological workflows. Consequently, while our findings establish a robust benchmark within our context, further validation in diverse international cohorts is necessary to determine if these specific numeric thresholds require adjustment for Western centers. Third, variations in surgical technique and pathological processing protocols could have influenced nodal yield independent of dissection adequacy. Finally, while our follow-up period is sufficient for initial survival analysis, longer-term data are needed to confirm the durability of the survival benefit associated with meeting these LNY thresholds, particularly in the context of late recurrences or immunotherapeutic late effects.

In conclusion, this development and validation study establishes stratified, procedure-specific LNY thresholds for neck dissection following NICT in locally advanced OSCC. Achieving a yield of ≥20 nodes in unilateral dissection or an average of ≥18 nodes per side in bilateral dissection is independently associated with significantly improved survival and an acceptable safety profile. These evidence-based benchmarks provide surgeons and multidisciplinary teams with a tangible, quantitative metric for assessing surgical quality and guiding postoperative adjuvant therapy decisions in the evolving treatment landscape of NICT. Its integration into clinical practice may help standardize surgical expectations and optimize oncologic outcomes for patients with locally advanced OSCC.

## Data Availability

The original contributions presented in the study are included in the article/**Supplementary Material**. Further inquiries can be directed to the corresponding author.
