# Supplementary material for: Stratified lymph node yield thresholds after neoadjuvant immunochemotherapy: a surgical benchmark for survival in oral squamous cell carcinoma
Source: Front Immunol. 2026 Jun 3;17:1782877. doi: 10.3389/fimmu.2026.1782877 (PMC13272155; doi:10.3389/fimmu.2026.1782877)
Supplement: Supplementary file 1 [file Table1.doc]

****Supplementary Table 1: Baseline Characteristics of the Training and Validation Cohorts****

| **Characteristic** | **Training Cohort (n=256)** | **Validation Cohort (n=199)** | **p** |
| --- | --- | --- | --- |
| ****Demographics**** |  |  |  |
| Age, median (IQR) | 62 (55-68) | 60 (54-67) | 0.215 |
| Sex, n (%) |  |  |  |
| Male | 185 (72.3) | 142 (71.4) | 0.830 |
| Female | 71 (27.7) | 57 (28.6) |  |
| BMI, median (IQR) | 20.1 (18.3-22.5) | 20.5 (18.5-22.8) | 0.342 |
| ****Clinical History**** |  |  |  |
| Smoking Status, n (%) |  |  |  |
| Never | 95 (37.1) | 80 (40.2) | 0.508 |
| Current/Former | 161 (62.9) | 119 (59.8) |  |
| Alcohol Status, n (%) |  |  |  |
| Never | 120 (46.9) | 98 (49.2) | 0.617 |
| Current/Former | 136 (53.1) | 101 (50.8) |  |
| ****Tumor Characteristics**** |  |  |  |
| Tumor Subsite, n (%) |  |  |  |
| Tongue | 110 (43.0) | 85 (42.7) | 0.956 |
| Buccal Mucosa | 45 (17.6) | 35 (17.6) |  |
| Floor of Mouth | 52 (20.3) | 40 (20.1) |  |
| Gingiva | 32 (12.5) | 26 (13.1) |  |
| Other | 17 (6.6) | 13 (6.5) |  |
| Clinical T Stage, n (%) |  |  |  |
| cT2 | 102 (39.8) | 78 (39.2) | 0.892 |
| cT3 | 98 (38.3) | 78 (39.2) |  |
| cT4 | 56 (21.9) | 43 (21.6) |  |
| Clinical N Stage, n (%) |  |  |  |
| cN0 | 88 (34.4) | 67 (33.7) | 0.874 |
| cN1 | 78 (30.5) | 63 (31.7) |  |
| cN2 | 75 (29.3) | 58 (29.1) |  |
| cN3 | 15 (5.9) | 11 (5.5) |  |
| ****Treatment Details**** |  |  |  |
| NICT Cycles, median (IQR) | 3 (2-4) | 3 (2-4) | 0.754 |
| Type of Neck Dissection, n (%) |  |  |  |
| Unilateral (Group Un) | 158 (61.7) | 122 (61.3) | 0.932 |
| Bilateral (Group Bi) | 98 (38.3) | 77 (38.7) |  |
| ****Pathological Data**** |  |  |  |
| Pathological T Stage (ypT), n (%) |  |  |  |
| ypT0/is | 75 (29.3) | 55 (27.6) | 0.899 |
| ypT1 | 52 (20.3) | 42 (21.1) |  |
| ypT2 | 68 (26.6) | 55 (27.6) |  |
| ypT3 | 38 (14.8) | 28 (14.1) |  |
| ypT4 | 23 (9.0) | 19 (9.5) |  |
| Pathological N Stage (ypN), n (%) |  |  |  |
| ypN0 | 182 (71.1) | 141 (70.9) | 0.970 |
| ypN1 | 42 (16.4) | 34 (17.1) |  |
| ypN2 | 28 (10.9) | 21 (10.6) |  |
| ypN3 | 4 (1.6) | 3 (1.5) |  |
| Lymphovascular Invasion (LVI), n (%) | 11 (4.3) | 8 (4.0) | 0.873 |
| Perineural Invasion (PNI), n (%) | 14 (5.5) | 11 (5.5) | 1.000 |
| ****Pathological Response, n (%)**** |  |  |  |
| pCR | 75 (29.3) | 55 (27.6) | 0.699 |
| mPR but not pCR | 104 (40.6) | 77 (38.7) | 0.682 |
| Non-mPR | 77 (30.1) | 67 (33.7) | 0.421 |
| ****Lymph Node Yield**** |  |  |  |
| Group Un: Total LNs, median (IQR) | 22 (9-45) | 24 (9-40) | 0.456 |
| Group Bi: Avg LNs/Side, median (IQR) | 20 (8-31) | 20 (8-36) | 0.311 |
| ****Adjuvant Therapy, n (%)**** | 256 (100.0) | 199 (100.0) | 1.000 |
| Radiotherapy alone | 128 (50.0) | 102 (51.3) | 0.798 |
| Concurrent Chemoradiotherapy | 128 (50.0) | 97 (48.7) |  |
