# Supplementary material for: Stratified lymph node yield thresholds after neoadjuvant immunochemotherapy: a surgical benchmark for survival in oral squamous cell carcinoma
Source: Front Immunol. 2026 Jun 3;17:1782877. doi: 10.3389/fimmu.2026.1782877 (PMC13272155; doi:10.3389/fimmu.2026.1782877)
Supplement: Supplementary file 2 [file Table2.doc]

### ****Supplementary Table 2: Cox Proportional Hazards Analysis for DFS in Group Un (Unilateral)****

| **Variable** | **Category** | **Univariable** |  | **Multivariable** |  |
| --- | --- | --- | --- | --- | --- |
|  |  | HR (95% CI) | p | aHR (95% CI) | p |
| ****LND Adequacy**** | Inadequate-Un LND (<20) | 2.34 (1.51–3.62) | <0.001 | 2.18 (1.35–3.52) | 0.001 |
| ****Age**** | ≥62 vs. <62 years | 1.28 (0.85–1.94) | 0.237 | 1.25 (0.81–1.92) | 0.312 |
| ****Sex**** | Male vs. Female | 1.15 (0.73–1.81) | 0.548 | 1.10 (0.69–1.76) | 0.690 |
| ****BMI**** | ≥20.1 vs. <20.1 kg/m² | 0.92 (0.61–1.38) | 0.678 | 0.95 (0.62–1.45) | 0.810 |
| ****Smoking Status**** | Current/Former vs. Never | 1.22 (0.80–1.86) | 0.356 | 1.18 (0.77–1.82) | 0.448 |
| ****Alcohol Status**** | Current/Former vs. Never | 1.14 (0.75–1.73) | 0.545 | 1.11 (0.73–1.70) | 0.626 |
| ****Tumor Subsite**** | Non-tongue vs. Tongue | 1.25 (0.83–1.88) | 0.287 | 1.22 (0.80–1.86) | 0.355 |
| ****Clinical T Stage**** | cT3/4 vs. cT2 | 1.42 (0.94–2.14) | 0.098 | 1.36 (0.89–2.08) | 0.154 |
| ****Clinical N Stage**** | cN+ vs. cN0 | 1.48 (0.95–2.31) | 0.083 | 1.42 (0.90–2.24) | 0.131 |
| ****Pathological T Stage**** | ypT3/4 vs. ypT0-2 | 1.82 (1.20–2.76) | 0.005 | 1.62 (1.06–2.48) | 0.027 |
| ****Pathological N Stage**** | ypN+ vs. ypN0 | 2.05 (1.36–3.09) | <0.001 | 1.82 (1.19–2.78) | 0.006 |
| ****LVI**** | Present vs. Absent | 2.28 (1.16–4.49) | 0.017 | 2.01 (1.01–4.01) | 0.047 |
| ****PNI**** | Present vs. Absent | 1.65 (0.78–3.51) | 0.191 | 1.48 (0.69–3.19) | 0.312 |
| ****Pathological Response**** | Non-mPR vs. mPR/pCR | 1.88 (1.24–2.84) | 0.003 | 1.70 (1.11–2.60) | 0.015 |
| ****Level Ⅳ/Ⅴ resect**** | No vs. Yes | 1.65 (0.58-3.34) | 0.319 | 1.90 (0.68-3.43) | 0.278 |
| ****NICT Cycles**** | ≥3 vs. <3 | 0.96 (0.64–1.45) | 0.852 | 0.98 (0.64–1.50) | 0.931 |
| ****Adjuvant Therapy**** | CRT vs. RT | 0.79 (0.53–1.19) | 0.262 | 0.82 (0.54–1.25) | 0.358 |
| ****Major Complications**** | Clavien-Dindo ≥III vs. <III | 1.58 (0.90–2.77) | 0.113 | 1.43 (0.81–2.55) | 0.217 |

****Notes:**** HR = Hazard Ratio; aHR = adjusted Hazard Ratio; Bold indicates statistical significance (p<0.05)
