# Supplementary material for: Stratified lymph node yield thresholds after neoadjuvant immunochemotherapy: a surgical benchmark for survival in oral squamous cell carcinoma
Source: Front Immunol. 2026 Jun 3;17:1782877. doi: 10.3389/fimmu.2026.1782877 (PMC13272155; doi:10.3389/fimmu.2026.1782877)
Supplement: Supplementary file 3 [file Table3.doc]

### ****Supplementary Table 3: Cox Proportional Hazards Analysis for DFS in Group Bi (Bilateral)****

| **Variable** | **Category** | **Univariable** |  | **Multivariable** |  |
| --- | --- | --- | --- | --- | --- |
|  |  | HR (95% CI) | p | aHR (95% CI) | p |
| ****LND Adequacy**** | Inadequate-Bi LND (<18/side) | 2.28 (1.24–4.19) | 0.008 | 2.05 (1.10–3.82) | 0.024 |
| ****Age**** | ≥60 vs. <60 years | 1.35 (0.77–2.37) | 0.297 | 1.30 (0.74–2.30) | 0.364 |
| ****Sex**** | Male vs. Female | 1.28 (0.67–2.43) | 0.458 | 1.25 (0.65–2.39) | 0.506 |
| ****BMI**** | ≥20.5 vs. <20.5 kg/m² | 0.85 (0.48–1.50) | 0.574 | 0.88 (0.49–1.57) | 0.659 |
| ****Smoking Status**** | Current/Former vs. Never | 1.38 (0.77–2.48) | 0.277 | 1.34 (0.74–2.42) | 0.331 |
| ****Alcohol Status**** | Current/Former vs. Never | 1.18 (0.67–2.09) | 0.568 | 1.15 (0.65–2.05) | 0.629 |
| ****Tumor Subsite**** | Non-tongue vs. Tongue | 1.42 (0.80–2.51) | 0.230 | 1.37 (0.77–2.44) | 0.285 |
| ****Clinical T Stage**** | cT3/4 vs. cT2 | 1.52 (0.86–2.70) | 0.150 | 1.46 (0.82–2.62) | 0.199 |
| ****Clinical N Stage**** | cN+ vs. cN0 | 1.58 (0.85–2.95) | 0.146 | 1.51 (0.80–2.85) | 0.203 |
| ****Pathological T Stage**** | ypT3/4 vs. ypT0-2 | 1.82 (1.02–3.24) | 0.043 | 1.65 (0.91–2.98) | 0.099 |
| ****Pathological N Stage**** | ypN+ vs. ypN0 | 2.18 (1.23–3.87) | 0.008 | 1.95 (1.08–3.51) | 0.026 |
| ****LVI**** | Present vs. Absent | 2.65 (1.16–6.06) | 0.021 | 2.28 (0.98–5.29) | 0.055 |
| ****PNI**** | Present vs. Absent | 1.78 (0.69–4.62) | 0.234 | 1.58 (0.60–4.15) | 0.355 |
| ****Pathological Response**** | Non-mPR vs. mPR/pCR | 1.96 (1.10–3.48) | 0.022 | 1.78 (0.99–3.19) | 0.053 |
| ****Level Ⅳ/Ⅴ resect**** | No vs. Yes | 1.81 (0.75-3.45) | 0.389 | 1.89 (0.68-3.56) | 0.234 |
| ****NICT Cycles**** | ≥3 vs. <3 | 0.91 (0.51–1.62) | 0.745 | 0.93 (0.52–1.67) | 0.810 |
| ****Adjuvant Therapy**** | CRT vs. RT | 0.73 (0.41–1.30) | 0.285 | 0.75 (0.42–1.35) | 0.339 |
| ****Major Complications**** | Clavien-Dindo ≥III vs. <III | 1.82 (0.90–3.69) | 0.096 | 1.68 (0.82–3.44) | 0.157 |

****Notes:**** HR = Hazard Ratio; aHR = adjusted Hazard Ratio; Bold indicates statistical significance (p<0.05)
