# Supplementary material for: Stratified lymph node yield thresholds after neoadjuvant immunochemotherapy: a surgical benchmark for survival in oral squamous cell carcinoma
Source: Front Immunol. 2026 Jun 3;17:1782877. doi: 10.3389/fimmu.2026.1782877 (PMC13272155; doi:10.3389/fimmu.2026.1782877)
Supplement: Supplementary file 4 [file Table4.doc]

****Supplementary Table 4: Sensitivity Analyses of Lymph Node Dissection (LND) Adequacy and Survival Outcomes****

| **Sensitivity Analysis** | **Method / Approach** | **Outcome** | **HR(95% CI)** | **p** | **Results** |
| --- | --- | --- | --- | --- | --- |
| ****Pooled analysis with surgical extent**** | Multivariable Cox model including surgical extent as covariate | OS | 2.36 (1.61–3.45) | <0.001 | ****Consistent**** |
|  |  | DFS | 2.12 (1.47–3.06) | <0.001 | ****Consistent**** |
| ****Bilateral cases: total LND threshold**** | Optimal cut-point for total LND (instead of average per side) | OS  (Bi, total LND <36) | 2.18 (1.14–4.16) | 0.018 | ****Consistent**** |
|  |  | DFS  (Bi, total LND <36) | 2.01 (1.08–3.74) | 0.027 | ****Consistent**** |
| ****Missing data handling**** | Complete-case vs. multiple imputation comparison | OS  (Group Un, aHR) | 2.42 vs. 2.40 | <0.001 (both) | ****Consistent**** |
|  |  | OS  (Group Bi, aHR) | 2.29 vs. 2.27 | 0.012 vs. 0.013 | ****Consistent**** |
